# Supplementary material for: Pyruvate Dehydrogenase Kinase 1 inhibition mediated oxidative phosphorylation enhancement in cartilage promotes osteoarthritis progression
Source: BMC Musculoskelet Disord. 2023 Jul 20;24:597. doi: 10.1186/s12891-023-06585-6 (PMC10357736; doi:10.1186/s12891-023-06585-6)
Supplement: Supplementary file 1 — Additional file 1. [file 12891_2023_6585_MOESM1_ESM.zip › KEGG.Gsea.1653622432464/KEGG_DRUG_METABOLISM_OTHER_ENZYMES.html]

Details for gene set KEGG\_DRUG\_METABOLISM\_OTHER\_ENZYMES[GSEA]

|  || Dataset | 55457\_collapsed\_to\_symbols.cls.cls #OA\_versus\_normal.cls.cls #OA\_versus\_normal\_repos |
| Phenotype | cls.cls#OA\_versus\_normal\_repos |
| Upregulated in class | OA |
| GeneSet | KEGG\_DRUG\_METABOLISM\_OTHER\_ENZYMES |
| Enrichment Score (ES) | 0.2807062 |
| Normalized Enrichment Score (NES) | 1.0720947 |
| Nominal p-value | 0.34114584 |
| FDR q-value | 0.50258034 |
| FWER p-Value | 1.0 |
Table: GSEA Results Summary

  

Fig 1: Enrichment plot: KEGG\_DRUG\_METABOLISM\_OTHER\_ENZYMES      
 Profile of the Running ES Score & Positions of GeneSet Members on the Rank Ordered List

  

| SYMBOL | TITLE | RANK IN GENE LIST | RANK METRIC SCORE | RUNNING ES | CORE ENRICHMENT || 1 | TPMT | thiopurine S-methyltransferase [Source:HGNC Symbol;Acc:HGNC:12014] | 54 | 0.981 | 0.0775 | Yes |
| 2 | NAT1 | N-acetyltransferase 1 [Source:HGNC Symbol;Acc:HGNC:7645] | 139 | 0.870 | 0.1435 | Yes |
| 3 | DPYS | dihydropyrimidinase [Source:HGNC Symbol;Acc:HGNC:3013] | 509 | 0.656 | 0.1695 | Yes |
| 4 | UGT2B4 | UDP glucuronosyltransferase family 2 member B4 [Source:HGNC Symbol;Acc:HGNC:12553] | 566 | 0.634 | 0.2180 | Yes |
| 5 | GUSB | glucuronidase beta [Source:HGNC Symbol;Acc:HGNC:4696] | 904 | 0.541 | 0.2369 | Yes |
| 6 | CES1 | carboxylesterase 1 [Source:HGNC Symbol;Acc:HGNC:1863] | 918 | 0.537 | 0.2807 | Yes |
| 7 | UGT2B17 | UDP glucuronosyltransferase family 2 member B17 [Source:HGNC Symbol;Acc:HGNC:12547] | 2050 | 0.356 | 0.2225 | No |
| 8 | UGT2A3 | UDP glucuronosyltransferase family 2 member A3 [Source:HGNC Symbol;Acc:HGNC:28528] | 2854 | 0.265 | 0.1821 | No |
| 9 | IMPDH2 | inosine monophosphate dehydrogenase 2 [Source:HGNC Symbol;Acc:HGNC:6053] | 2867 | 0.264 | 0.2032 | No |
| 10 | TK1 | thymidine kinase 1 [Source:HGNC Symbol;Acc:HGNC:11830] | 2909 | 0.259 | 0.2216 | No |
| 11 | UMPS | uridine monophosphate synthetase [Source:HGNC Symbol;Acc:HGNC:12563] | 2936 | 0.257 | 0.2410 | No |
| 12 | UGT1A8 | UDP glucuronosyltransferase family 1 member A8 [Source:HGNC Symbol;Acc:HGNC:12540] | 3301 | 0.221 | 0.2311 | No |
| 13 | TYMP | thymidine phosphorylase [Source:HGNC Symbol;Acc:HGNC:3148] | 3588 | 0.195 | 0.2251 | No |
| 14 | CES2 | carboxylesterase 2 [Source:HGNC Symbol;Acc:HGNC:1864] | 3689 | 0.186 | 0.2329 | No |
| 15 | ITPA | inosine triphosphatase [Source:HGNC Symbol;Acc:HGNC:6176] | 3751 | 0.181 | 0.2433 | No |
| 16 | DPYD | dihydropyrimidine dehydrogenase [Source:HGNC Symbol;Acc:HGNC:3012] | 3830 | 0.173 | 0.2517 | No |
| 17 | GMPS | guanine monophosphate synthase [Source:HGNC Symbol;Acc:HGNC:4378] | 4498 | 0.113 | 0.2092 | No |
| 18 | CYP3A43 | cytochrome P450 family 3 subfamily A member 43 [Source:HGNC Symbol;Acc:HGNC:17450] | 4698 | 0.096 | 0.2018 | No |
| 19 | UGT1A10 | UDP glucuronosyltransferase family 1 member A10 [Source:HGNC Symbol;Acc:HGNC:12531] | 4850 | 0.083 | 0.1970 | No |
| 20 | NAT2 | N-acetyltransferase 2 [Source:HGNC Symbol;Acc:HGNC:7646] | 4994 | 0.074 | 0.1920 | No |
| 21 | HPRT1 | hypoxanthine phosphoribosyltransferase 1 [Source:HGNC Symbol;Acc:HGNC:5157] | 6954 | -0.076 | 0.0461 | No |
| 22 | UGT2B11 | UDP glucuronosyltransferase family 2 member B11 [Source:HGNC Symbol;Acc:HGNC:12545] | 7270 | -0.100 | 0.0299 | No |
| 23 | TK2 | thymidine kinase 2 [Source:HGNC Symbol;Acc:HGNC:11831] | 7468 | -0.116 | 0.0243 | No |
| 24 | CYP2A6 | cytochrome P450 family 2 subfamily A member 6 [Source:HGNC Symbol;Acc:HGNC:2610] | 7849 | -0.144 | 0.0067 | No |
| 25 | CYP3A5 | cytochrome P450 family 3 subfamily A member 5 [Source:HGNC Symbol;Acc:HGNC:2638] | 8272 | -0.177 | -0.0113 | No |
| 26 | CDA | cytidine deaminase [Source:HGNC Symbol;Acc:HGNC:1712] | 8324 | -0.180 | -0.0003 | No |
| 27 | XDH | xanthine dehydrogenase [Source:HGNC Symbol;Acc:HGNC:12805] | 8366 | -0.183 | 0.0118 | No |
| 28 | CYP3A7 | cytochrome P450 family 3 subfamily A member 7 [Source:HGNC Symbol;Acc:HGNC:2640] | 9024 | -0.234 | -0.0198 | No |
| 29 | UGT2A1 | UDP glucuronosyltransferase family 2 member A1 complex locus [Source:HGNC Symbol;Acc:HGNC:12542] | 9451 | -0.269 | -0.0305 | No |
| 30 | UPB1 | beta-ureidopropionase 1 [Source:HGNC Symbol;Acc:HGNC:16297] | 9609 | -0.284 | -0.0190 | No |
| 31 | CYP3A4 | cytochrome P450 family 3 subfamily A member 4 [Source:HGNC Symbol;Acc:HGNC:2637] | 10193 | -0.340 | -0.0360 | No |
| 32 | UCK2 | uridine-cytidine kinase 2 [Source:HGNC Symbol;Acc:HGNC:12562] | 10481 | -0.371 | -0.0274 | No |
| 33 | CYP2A13 | cytochrome P450 family 2 subfamily A member 13 [Source:HGNC Symbol;Acc:HGNC:2608] | 10732 | -0.397 | -0.0138 | No |
| 34 | IMPDH1 | inosine monophosphate dehydrogenase 1 [Source:HGNC Symbol;Acc:HGNC:6052] | 11023 | -0.433 | -0.0002 | No |
| 35 | UCKL1 | uridine-cytidine kinase 1 like 1 [Source:HGNC Symbol;Acc:HGNC:15938] | 11515 | -0.503 | 0.0035 | No |
| 36 | UPP1 | uridine phosphorylase 1 [Source:HGNC Symbol;Acc:HGNC:12576] | 12013 | -0.595 | 0.0145 | No |
| 37 | UGT2B15 | UDP glucuronosyltransferase family 2 member B15 [Source:HGNC Symbol;Acc:HGNC:12546] | 12225 | -0.652 | 0.0525 | No |
Table: GSEA details [plain text format]

  

Fig 2: KEGG\_DRUG\_METABOLISM\_OTHER\_ENZYMES      
 Blue-Pink O' Gram in the Space of the Analyzed GeneSet

  

Fig 3: KEGG\_DRUG\_METABOLISM\_OTHER\_ENZYMES: Random ES distribution      
 Gene set null distribution of ES for **KEGG\_DRUG\_METABOLISM\_OTHER\_ENZYMES**
